# Human immunodeficiency virus knowledge, attitudes, and testing behaviors among adults in Saudi Arabia: a cross-sectional analysis to inform public health strategies

**DOI:** 10.3389/fpubh.2026.1830405

**Published:** 2026-06-09

**Authors:** Yahya Mohamed Shabi, Abdelwahid Saeed Ali, Abdulah Jarboa Alqahtani, Amal Khairan Alamri, Nada Abdullah Alharbi, Thamir Saad Alsaeed

**Affiliations:** 1 Department of Microbiology and Clinical Parasitology, College of Medicine, King Khalid University, Abha, Saudi Arabia; 2 Department of Adult Infectious Diseases, Armed Forces Hospital Southern Region (AFHSR), Khamis Mushait, Saudi Arabia; 3 Department of Basic Medical Sciences, College of Medicine, Princess Nourah bint Abdulrahman University, Riyadh, Saudi Arabia; 4 Department of Biology and Immunology, College of Medicine, Qassim University, Riyadh, Saudi Arabia

**Keywords:** HIV, knowledge, attitudes, testing, gaps, Saudi Arabia

## Abstract

**Introduction:**

The Middle East and North Africa (MENA) region has experienced a notable increase in HIV infections in recent years, with limited epidemiological and behavioral data from Gulf Cooperation Council (GCC) countries, including Saudi Arabia. This study aimed to address the critical knowledge gaps by examining HIV-related knowledge, attitudes, and testing behaviors among adults in Saudi Arabia to inform culturally appropriate public health interventions.

**Methodology:**

A cross-sectional survey was conducted among adults from various regions of Saudi Arabia. Data were collected using a validated electronic Arabic-language questionnaire that assessed socio-demographic characteristics, objective HIV knowledge, self-perceived knowledge, attitudes towards people living with HIV (PLWHIV), history of testing, exposure to health education, and perceptions of societal stigma. A total of 475 eligible respondents were considered in the analyses.

**Results:**

The overall mean HIV knowledge score was 6.62 ± 2.44. Although 364 (76.6%) of participants identified the primary modes of HIV transmission, 209 (44.0%) perceived HIV as a fatal disease. Furthermore, 258 (54.3%) of respondents perceived Saudi society as “not accepting” of people living with HIV (PLWHIV), and 285 (60.0%) considered discussions about HIV to be culturally sensitive. The rate of voluntary HIV testing was low. The predominant reason cited was “testing was unnecessary”. However, among those who had never been tested, 177 (54.5%) expressed willingness to undergo testing if it were provided free of charge and confidential. Majorities of respondents supported the integration of HIV-related information into educational curricula (93.5%). Higher educational attainment (*p* = 0.003), prior HIV testing (*p* < 0.001), and exposure to health education (*p* < 0.001) were significantly associated with higher HIV knowledge scores. Interestingly, 99 (66.0%) of individuals who had previously undergone HIV testing, compared with 159 (48.9%) of those who had never been tested, perceived society as non-accepting of PLWHIV.

**Discussion:**

This study confirms substantial gaps in comprehensive HIV knowledge, persistent societal stigma, and low uptake of voluntary HIV testing among adults in Saudi Arabia. Despite these challenges, there is a considerable public support for expanding HIV education and improving access to testing services. The findings highlight the urgent need for culturally sensitive, evidence-based public health strategies that address knowledge deficits, reduce stigma, and promote widely accessible HIV testing services. Strengthening these efforts is essential to accelerate Saudi Arabia’s progress toward achieving the UNAIDS 95-95-95 targets.

## Introduction

HIV/AIDS has remained a major global public health challenge since the early 1980s. In 2024, approximately an estimated 630,000 deaths were attributed to HIV-related causes worldwide, and an estimated 1.3 million individuals newly acquired HIV infection (1). Notably, the Middle East and North Africa (MENA) region is among the regions globally exhibiting the most rapidly increasing HIV incidences and expanding epidemics (2, 3). This increase is primarily attributable to ongoing transmission of the virus among key populations at elevated risk and young adults aged 15–24 years, further, compounded by sociocultural barriers, including stigma, discrimination, and limited access to comprehensive HIV prevention and harm-reduction services (3–5). The Joint United Nations Program on HIV/AIDS (UNAIDS) has established the ambitious 95–95-95 targets for 2025, which aim to ensure that 95% of people living with HIV are diagnosed, 95% of those diagnosed initiate and sustain antiretroviral therapy (ART), and 95% of individuals receiving ART achieve durable viral suppression (6). However, epidemiological data from Gulf Cooperation Council (GCC) member states, including Saudi Arabia, remain limited, with substantial heterogeneity in HIV-related policies, surveillance systems, and program implementation across these countries.

Despite the relatively low HIV prevalence in Saudi Arabia compared with global estimates, multiple studies have identified substantial gaps in HIV-related knowledge, attitudes, and testing practices among adults (7–9). Furthermore, available estimates suggested that HIV-related mortality in Saudi Arabia remains relatively low, with approximately 200 AIDS-related deaths reported in 2024 (10). The HIV-knowledge gaps in Saudi Arabia are closely linked to prevailing sociocultural and religious norms, which may contribute to delayed diagnosis, underutilization of testing services, and barriers to effective prevention and treatment. A primary concern is insufficient comprehensive knowledge of HIV transmission dynamics, with limited understanding of established modes of transmission representing a major barrier to timely testing and risk-reduction behaviors (8). Although most adults in Saudi Arabia can accurately identify primary HIV transmission routes, such as unprotected sexual contact and sharing contaminated needles, persistent misconceptions remain regarding casual modes of transmission, including sharing utensils, toilet seats, or insect bites. This indicates the need for targeted public health education campaigns that extend beyond basic transmission facts to actively dispel entrenched myths. Moreover, there is a marked deficiency in awareness regarding HIV co-infections, the availability and use of post-exposure prophylaxis (PEP), and the understanding that HIV is a manageable chronic condition with effective ART therapies, rather than inevitably fatal disease (7, 11–13). These knowledge gaps contribute significantly to fear, stigma, and reluctance to seek testing or care. Alarmingly, similar gaps have been observed among healthcare professionals, which may adversely affect the quality and appropriateness of HIV-related services provided (12, 14).

A second critical gap relates to attitudinal barriers and stigma. High levels of stigma and discrimination toward people living with HIV (PLWHIV) represent a substantial impediment to effective testing, treatment and prevention efforts. Empirical studies consistently document negative attitudes within the general population, as well as among some healthcare providers (15–18). Such stigma is frequently driven by fear of contagion and sociocultural perceptions that associate HIV infection with socially or religiously “inappropriate” behaviors, or interpret it as a form of moral retribution. These perceptions foster moral judgment, social distancing, and diminished empathy toward PLWHIV. Consequently, a proportion of individuals report reluctance to interact with or support PLWHIV, with some endorsing social exclusion. This entrenched stigma contributes to underreporting of cases, delayed testing, and reduced engagement in care due to fear of social repercussions and discrimination. These concerns may be particularly pronounced among married women, who may not perceive themselves at risk of infection and may face additional sociocultural barriers to seeking HIV testing and care (18, 19). HIV testing in Saudi Arabia is conducted at multiple healthcare levels, including primary healthcare centers, hospitals, blood banks, antenatal screening services, premarital screening programs, and specialized infectious disease clinics. HIV screening is also routinely performed for blood and organ donors, selected high-risk groups, and expatriates applying for residency or employment permits (20). The third gap in Saudi Arabia pertains to testing behavior, as low rates of voluntary HIV testing remain a critical public health concern. A substantial proportion of the population- particularly younger individuals- have never undergone HIV testing, largely due to a low perceived personal risk of infection (7, 14, 21). Individuals who strongly adhere to religious values may perceive themselves as not being at risk, while married women often assume spousal fidelity, contributing to delayed diagnosis. Consequently, HIV infection is frequently identified only during mandatory pre-marital screening or following the onset of illness in a child. Additionally, fear of a positive test result and its potential social ramifications- including stigma, and concerns regarding breaches of confidentiality within healthcare settings- serves as a significant barrier to voluntary testing. The predominant reliance on mandatory screening programs highlights a critical gap in proactive, self-initiated health-seeking behavior and individual risk awareness (13, 22, 23).

The fourth gap relates to underlying contributory factors. These factors are compounded by conservative sociocultural norms that heavily influence perceptions of HIV/AIDS, often associating the disease with socially taboo behaviors. Such norms restrict open discourse and limit the provision of comprehensive, evidence-based education (24). Although recent awareness campaigns have demonstrated some progress, there remains a clear need for sustained, culturally appropriate, and targeted educational interventions. These initiatives should aim to address specific knowledge deficits, correct persistent misconceptions, and actively reduce stigmatizing attitudes among both the general population and healthcare professionals (21, 23, 25). In addition, the recognized paucity of comprehensive research on HIV/AIDS in Saudi Arabia further hinders the development and implementation of context-specific, evidence-based policies and interventions. This study aims to examine the extent of these identified gaps within Saudi society by assessing HIV-related knowledge, attitudes, and testing behaviors among adults in Saudi Arabia. By systematically, evaluating these domains, the study seeks to generate evidence that can inform targeted interventions. The findings will be instrumental in guiding the development of culturally appropriate, evidence-based public health strategies aimed at promoting early diagnosis, reducing stigma, and supporting Saudi Arabia’s progress toward achieving the UNAIDS 95–95-95 targets.

## Methods

### Participants’ selection and enrollment

Participants were enrolled using an electronic questionnaire-based recruitment process. The questionnaire link was distributed electronically via email, social media platforms, and messaging applications. Individuals who met the study inclusion criteria were invited to participate in the study. The purpose, objectives, and procedures of the study to potential participants were well defined and explained in the structured- questionnaire specifically designed for the study. The sampling frame was clearly defined, and eligible participants were selected according to predefined inclusion and exclusion criteria to minimize selection bias and enhance the generalizability of the findings. Inclusion criteria for participation included: Individuals aged 18 years and above, residents of the selected area of study, individuals able to read and understand the questionnaire language, respondents who are willing to participate voluntary, and those who responded to the informed consent before participations were included. On another hand, respondents below 18 years of age, declined to give informed consent, had incomplete questionnaire responses, and those unable to understand the questionnaire due to language difficulties were excluded from the study. Participation was voluntary, and participants could withdraw from the study at any stage before submission of the questionnaire.

The electronic questionnaire was self-administered and used to assess participants’ HIV knowledge, attitudes, and testing behavior. To ensure confidentiality and privacy, no personally identifiable information was collected. Only responses from participants who completed the questionnaire and satisfied the inclusion criteria were included in the final analysis. To minimize the selection bias, a consecutive sampling method was applied, in which all eligible individuals who accessed the electronic questionnaire during the study period were invited to participate until the required sample size was reached. Additionally, the standardized inclusion and exclusion criteria were applied uniformly to all participants to maintain consistency throughout the enrollment process. The same electronic questionnaire, instructions, and consent procedure were used for all respondents to reduce procedural variation. In addition, participation was anonymous and voluntary, which encouraged honest responses regarding HIV knowledge and testing behavior.

After all participants completed the questionnaire, a total of 475 participants from different regions of Saudi Arabia (including the Central, Southern, Western, Eastern, and Northern parts of the country) were recruited.

### Participants’ consent

Informed consent was obtained electronically from all participants prior to participation. Specifically, the first page of the online questionnaire included a detailed participant information statement outlining the study purpose, voluntary nature of participation, confidentiality of responses, and their right to withdraw at any time without any consequences. Participants were required to provide their consent by selecting an agreement option before proceeding to the survey questions. Completion and submission of the questionnaire were considered to constitute implied consent.

The study protocol, including the consent procedure, was reviewed and approved by the Research Ethics Committee of King Khalid University, Saudi Arabia (Approval No. ECM#2025–101).

### Instrument and data collection

The study instrument was a validated electronic questionnaire developed in Arabic and adapted to ensure cultural appropriateness and adopted as previously described by Al-Moziani et al. (26). The questionnaire comprised items assessing sociodemographic characteristics, factual knowledge of HIV transmission and treatment, self-perceived knowledge, attitudes toward people living with HIV (PLWHIV), previous HIV testing behavior, and willingness to undergo testing. In addition, participants were asked about their exposure to HIV-related health education, perceptions of societal attitudes toward HIV, and opinions regarding the expansion of access to HIV testing services.

### Knowledge score construction

The HIV Knowledge Score (KS) was constructed using responses to multiple factual items designed to assess participants’ understanding of HIV transmission, prevention, and treatment (27). The questionnaire included key knowledge domains such as modes of HIV transmission, misconceptions regarding casual contact, preventive strategies including condom use and safe sexual practices, and awareness of HIV testing and treatment availability. Each item was scored using a three-level response system to capture varying degrees of certainty and knowledge accuracy. Correct responses were assigned 2 points, reflecting accurate HIV-related knowledge. “Not sure” responses were assigned 1 point to differentiate uncertainty from clearly incorrect knowledge, while incorrect responses received 0 points. This approach allowed the scoring system to account for partial awareness and reduce the likelihood of equating uncertainty with misinformation. The individual item scores were summed to generate a composite HIV Knowledge Score, with a total possible score ranging from 0 to 10. Higher scores indicated greater HIV-related knowledge and awareness. The use of a composite score enabled quantitative assessment of participants’ overall HIV knowledge and facilitated comparison across demographic and behavioral variables. Furthermore, adoption of this previously published scoring methodology enhanced methodological consistency and comparability with earlier HIV knowledge studies.”

### Statistical analysis

Data were analyzed using *IBM SPSS* Statistics for Windows, version 26.0 (IBM Corp., Armonk, NY, United States). Descriptive statistics were computed to summarize participant’s demographic characteristics, HIV knowledge levels, attitudes, and testing behaviors. Categorical variables were presented as frequencies and percentages, whereas continuous variables, including the HIV knowledge score, were summarized using means and standard deviations (SD).

Inferential analyses were performed to examine differences in HIV knowledge scores across subgroups. Independent- samples *t*-tests were used to compare mean knowledge scores between dichotomous groups (e.g., gender, HIV testing history, receipt of health education). One-way analysis of variance (ANOVA) was applied for variables with more than two categories (e.g., education level). For statistically significant ANOVA results, *post hoc* pairwise comparisons were conducted using Tukey’s honestly significant difference (HSD) test. Homogeneity of variances was assessed with using Levene’s test, and appropriate adjustments were applied when assumption was violated. Associations between categorical variables (e.g., education level and HIV testing behavior) were examined using Fisher’s Exact Test due to small expected cell counts in some categories. A two-sided *p*-value of < 0.05 was considered statistically significant for all analyses.

## Results

### Sociodemographic characteristics of the participants

Table 1 summarizes the sociodemographic characteristics of the enrolled study participants (*N* = 475). The largest proportion of respondents was aged 30–40 years (202; 42.5%), followed by those aged 20–30 years (126; 26.5%). Participants aged 50 years and above represented the smallest age group (10.5%). Regarding gender distribution, males accounted for 262 (55.2%) of the sample, whereas females constituted 213 (44.8%). The vast majority of participants were Saudi nationals 455 (95.8%). In terms of educational attainment, most respondents held a university degree 353 (74.3%), while 79 (16.6%) had completed secondary education. Fewer than 10% of participants reported primary, intermediate, or postgraduate levels of education. With respect to regional distribution, the highest proportion of participants was from the southern region 186 (39.2%), followed by the central 131 (27.6%), eastern 62 (13.1%), western 59 (12.4%), and northern 37 (7.8%) regions of the country.

**Table 1:** Sociodemographic characteristics of the participants.

Variable	Category	No (%)*
Age (years)	20–30	126 (26.5)
	30–40	202 (42.5)
	40–50	97 (20.5)
	≥ 50	50 (10.5)
Gender	Male	262 (55.2)
	Female	213 (44.8)
Nationality	Saudi	455 (95.8)
	Non-Saudi	20 (4.2)
Education level	Primary	1 (0.2)
	Intermediate	6 (1.3)
	Secondary	79 (16.6)
	University	353 (74.3)
	Postgraduate	36 (7.6)
Region	Northern	37 (7.8)
	Southern	186 (39.1)
	Central	131 (27.6)
	Western	59 (12.4)
	Eastern	62 (13.1)

* Total No. of participants = 475.

### HIV knowledge among participants

The overall mean HIV knowledge score among participants was 6.62 ± 2.44. A substantial proportion of respondents (364, 76.6%) correctly identified the primary modes of HIV transmission. There was no marked difference in the proportion of participants who were able to distinguish between HIV and ADIS and those who were not. Regarding perception of HIV, the majority of respondents considered HIV to be a fatal disease. Additionally, 227 (47.8%) recognized condom use as an effective preventive measure against HIV transmission. In terms of self-assessed HIV knowledge, relatively similar proportions were observed across categories: 177 (37.3%) of participants demonstrated good knowledge, 170 (35.8%) demonstrated moderate knowledge, and 128 (26.9%) demonstrated limited knowledge to HIV (Table 2).

**Table 2:** HIV Knowledge, specific beliefs, and self-assessment.

Item/category	Category response	No (%) or Mean ±SD	Effect size (95% CI)	*p*value
Overall HIV Knowledge Score		6.62 ± 2.44		
Difference between HIV infection and AIDS	Yes	188 (39.6)	*V = 0.432 (95% CI: 0.345–0.516)*	<0.001*
	No	187 (39.4)		
	Not sure	100 (21.0)		
Awareness of primary transmission modes	Correct	364 (76.6)	*OR = 3.18 (95% CI: 1.82–5.57)*	<0.001*
	Wrong	111 (23.4)		
Belief: HIV is a fatal disease	Yes	209 (44.0)	*V = 0.167 (95% CI: 0.088–0.258)*	0.001*
	No	150 (31.6)		
	Not sure	116 (24.4)		
Awareness: HIV can be managed with treatment	Yes	297 (62.5)	*V = 0.320 (95% CI: 0.246–0.393)*	<0.001*
	No	65 (13.7)		
	Not sure	113 (23.8)		
General knowledge statements (correctly answered)	Correct	409 (85.9)	*OR = 2.63 (95% CI: 1.34–5.18)*	0.004*
	Wrong	67 (14.1)		
Using condom is a prevention method.	True	227 (47.8)		
	Untrue	248 (52.2)		
Self-assessment of HIV knowledge	Good knowledge	177 (37.3%)	*V = 0.460 (95% CI: 0.387–0.537)*	<0.001*
	Moderate knowledge	170 (35.8)		
	Limited knowledge	128 (26.9)		

HIV, human immunodeficiency virus; AIDS, acquired immunodeficiency syndrome; CI, confidence interval; OR, odds ratio; V, Cramér’s V; N/A, not applicable. All comparisons are between participants previously tested for HIV (*n* = 150) vs. those never tested (*n* = 325). *p < 0.05 considered statistically significant. Statistical tests: Pearson chi-square unless any expected cell count < 5; †*p*-values computed using Monte Carlo permutation test (10,000 iterations). Effect size: OR (odds ratio) reported for binary outcomes; Cramér’s V reported for polytomous outcomes (negligible < 0.10, weak 0.10–0.19, moderate 0.20–0.39, strong ≥ 0.40; Cohen, 1988). OR interpreted as odds of the event in previously-tested vs. never-tested participants (reference: never tested). 95 CI for OR computed by Woolf’s method. 95 CI for Cramér’s V estimated by bias-corrected bootstrap (2,000 resamples). Total No. of participants = 475.

### HIV testing behavior and societal perceptions among participants

Table 3 illustrates participants’ HIV testing behaviors and societal perception related to HIV. Among the total study population, only 150 (31.6%) reported having previously undergone HIV testing. Among those who had never been tested, more than half (177, 54.5%) expressed willingness to undergo testing if it was provided free of charge and ensured confidentiality. The commonly reported reason for not undergoing HIV test was the perception that there was no need for testing (248, 76.3%).

**Table 3:** HIV testing behavior, societal perceptions, and barriers.

Item/Category	Category response	No (%)	Effect size (95% CI)	*p*value
Ever taken HIV test?	Yes	150 (31.6)		
	No	325 (68.4)		
Willingness to take HIV test (among never tested, *n* = 325)	Yes ^+^	177 (54.5)	*N/A*	N/A
	Not sure	79 (24.3)		
	No	69 (21.2)		
Ever received health education about HIV/AIDS?	Yes	189 (39.8)	*OR = 3.89 (95% CI: 2.59–5.85)*	<0.001*
	No	286 (60.2)		
Perception of Saudi society’s acceptance of PLWHIV	Fully accepting	15 (3.2)	*V = 0.160 (95% CI: 0.095–0.258)*	0.005†
	Accepting with caution	113 (23.8)		
	Not sure	89 (18.7)		
	Not accepting	258 (54.3)		
Belief: Discussion HIV is culturally /religiously sensitive	Yes	285 (60.0)	*V = 0.120 (95% CI: 0.046–0.212)*	0.032*
	Not sure	84 (17.7)		
	No	106 (22.3)		
Belief: Families provide enough support to PLHIV	Yes	98 (20.6)	*V = 0.107 (95% CI: 0.038–0.203)*	0.065
	Not sure	222 (46.8)		
	No	155 (32.6)		
Reported reasons for not taking an HIV test (among never tested, *n* = 325)	Lack of time	51 (15.7)		
	Fear of community or close people’s reaction	9 (2.8)		
	Not knowing where to get tested	31 (9.5)		
	Financial reasons (cost of testing is high)	10 (3.1)		
	Belief that there is no need to get tested	248 (76.3)		
	I do not want to know	17 (5.2)		
	Other reasons	10 (3.1)		

HIV, human immunodeficiency virus; AIDS, acquired immunodeficiency syndrome; CI, confidence interval; OR, odds ratio; V, Cramér’s V; N/A, not applicable; PLWHIV = People living with HIV. All comparisons are between participants previously tested for HIV (*n* = 150) vs. those never tested (*n* = 325). *p < 0.05 considered statistically significant. Statistical tests: Pearson chi-square unless any expected cell count < 5; †*p*-values computed using Monte Carlo permutation test (10,000 iterations). Effect size: OR (odds ratio) reported for binary outcomes; Cramér’s V reported for polytomous outcomes (negligible < 0.10, weak 0.10–0.19, moderate 0.20–0.39, strong ≥ 0.40; Cohen, 1988). OR interpreted as odds of the event in previously-tested vs. never-tested participants (reference: never tested). 95 CI for OR computed by Woolf’s method. 95 CI for Cramér’s V estimated by bias-corrected bootstrap (2,000 resamples). Total No. of participants = 475. Willingness to test and reasons for not testing calculated from never-tested participants (*n* = 325). ^+^ Willing if test offered free and confidential. N/A, question asked only of never-tested participants; comparison not applicable.

A statistically significant difference was noted between participants who had previously received health education about HIV/AIDS and those who had not (*p* ≤ 0.05).

Regarding the perceptions, majority 258 (54.3%) of respondents perceived Saudi society as “not accepting” of people living with HIV (PLWHIV), and 285 (60%) considered discussions about HIV to be culturally or religiously sensitive.

### Attitudes toward HIV education and testing

The mean agreement score for HIV awareness programs was 4.55 ± 0.74, indicating a generally positive attitude toward such initiatives. An overwhelming majority of the respondents (444, 93.5%) supported the inclusion of HIV-related information in educational curricula. Additionally, 321 (67.6%) of participants strongly agreed with the implementation of HIV awareness programs. A considerable proportion of participants (318, 66.9%) were aware that HIV infection can be accurately detected through simple laboratory tests. Regarding stigma reduction, there was no significant variation among respondents in their belief that anonymous rapid HIV testing could help reduce stigma, with a mean agreement score of 3.90 ± 1.08. However, a statistically significant difference was observed among respondents regarding the belief that boarder HIV testing could increase uptake (Table 4).

**Table 4:** Attitudes toward HIV education, awareness programs, and access to diagnostic testing.

Item/category	Category response	No (%)	Effect size (95% CI)	*p* value
Support for including HIV-related information in the educational curricula	Yes	444 (93.5)	*OR = 0.97 (95% CI: 0.44–2.11)*	0.933
	No	31 (6.5)		
Extent of acceptance for HIV awareness programs	Strongly agree	321 (67.6)	*V = 0.066 (95% CI: 0.044–0.155)*	0.734†
	Agree	108 (22.7)		
	Neutral	36 (7.6)		
	Disagree	7 (1.5)		
	Strongly disagree	3 (0.6)		
Agreement score for awareness programs (mean ±SD)		4.55 ± 0.74		
Awareness: HIV can be detected by accurate and simple lab tests?	Yes	318 (66.9)	*V = 0.334 (95% CI: 0.266–0.402)*	<0.001*
	Not sure	83 (17.5)		
	No	74 (15.6)		
Support for random HIV testing in public places (e.g., malls, campaigns)	Yes	301 (63.4)	*V = 0.051 (95% CI: 0.013–0.156)*	0.538
	Not sure	73 (15.4)		
	No	101 (21.2)		
Belief: Anonymous rapid HIV testing would help reduce stigma	Strongly agree	181 (38.1)	*V = 0.112 (95% CI: 0.063–0.214)*	0.206†
	Agree	126 (26.5)		
	Neutral	121 (25.5)		
	Disagree	33 (7.0)		
	Strongly disagree	14 (2.9)		
Agreement score for anonymous testing reducing stigma (mean ±SD)		3.90 ± 1.08		
Belief: Broader HIV testing availability would increase uptake	Yes	337 (70.9)	*OR = 1.19 (95% CI: 0.77–1.83)*	0.475
	Not sure	101 (21.3)		
	No	37 (7.8)		

Total No. of participants = 475. Comparisons by HIV testing history (previously tested, *n* = 150 vs. never tested, *n* = 325). Chi-square used unless any expected cell count < 5; †*p*-values computed using Monte Carlo permutation test (10,000 iterations) when expected cell count < 5 in tables larger than 2 × 2. Fisher’s exact test not applicable to tables larger than 2 × 2. V = Cramér’s V effect size. OR, Odds Ratio (95% CI). *p < 0.05 considered statistically significant.

### HIV knowledge score across demographic, testing and societal perception groups

The analysis demonstrated that participants with higher educational attainment had significantly higher knowledge scores (KS) compared with those with lower levels of education (*p* = 0.003). In addition, participants who had previously undergone HIV testing exhibited significantly higher KS than those who had never been tested (*p* < 0.001). Exposures to health education was also significantly associated with higher HIV KS (Table 5).

**Table 5:** Comparison of HIV knowledge score across demographic groups and testing status.

Participant group	No.	Knowledge Score (mean± SD)	Test type	Test statistic	*p*-value
Education level					
Primary	1	4.00(−)	One way ANOVA	*F*(4,470) = *p* 0.15	0.003*
Intermediate	6	5.67 ± 1.21			
Secondary	79	5.81 ± 2.29			
University	353	6.73 ± 2.46			
Post Graduate	36	7.50 ± 2.29			
Gender					
Male	262	6.78 ± 2.53	T-test	t(473) = *p* 0.61	0.109
Female	213	6.42 ± 2.32			
HIV test history					
Never tested	325	6.01 ± 2.37	T-test	t(473) = 8.63	< 0.001*
Previously tested	150	7.94 ± 2.04			
Received health education					
Yes	189	7.60 ± 2.06	T-test	t(473) = 7.53	< 0.001*
No	286	5.97 ± 2.46			

SD, Standard Deviation; Total No. of participants = 475. *p < 0.05 considered statistically significant. For education levels: equal variance assumption was met. For HIV testing comparison: equal variances were not assumed (Levene’s *p* = 0.013).

Furthermore, a significantly greater proportion of participants with university-level education (122, 81.33%) reported having previously undergone HIV testing compared with participants with lower educational levels. Interestingly, 99 (66.0%) participants who had previously been tested for HIV perceived societal attitudes toward people living with HIV (PLWHIV) as non-accepting, whereas 159 (48.9%) who had never been tested, perceived attitudes toward PLWHIV as non-accepting (Table 6).

**Table 6:** Associations between education level, perception of societal acceptance, health education and HIV testing history.

Variable	History of HIV testing-never tested No. (%)	History of HIV testing-previously tested No. (%)	Total no. (%)
Education level			
Primary	1 (0.30)	0 (0.00)	1 (0.21)
Intermediate	6 (1.84)	0 (0.00)	6 (1.26)
Secondary	67 (20.61)	12 (8.00)	79 (16.63)
University	231 (71.10)	122 (81.33)	353 (74.32)
Post graduate	20 (6.15)	16 (10.67)	36 (7.58)
Perception of societal acceptance			
Not accepted	159 (48.92)	99 (66.00)	258 (54.32)
Not sure.	68 (20.92)	21 (14.00)	89 (18.73)
Accepted but with caution	87 (26.77)	26 (17.33)	113 (23.79)
Fully accepting	11 (3.39)	4 (2.67)	15 (3.16)
Received health education			
No	229 (70.46)	57 (38.00)	286 (60.21)
Yes	96 (29.54)	93 (62.00)	189 (39.79)

Fisher’s exact test, *p* < 0.05 considered statistically significant. Fisher’s Exact Test used due to small expected cell counts.

## Discussion

This cross-sectional study examined HIV knowledge, attitudes, and testing behaviors among adults in Saudi Arabia, providing important insights to inform targeted public health strategies. The findings highlight persistent gaps in HIV-related knowledge, significant attitudinal barriers largely driven by stigma, and relatively low rates of voluntary HIV testing. Collectively, these factors represent critical challenges to achieving the ambitious UNAIDS 95–95-95 targets for HIV diagnosis, treatment, and viral suppression in the region.

The overall mean HIV knowledge score of 6.62 ± 2.44 obtained in this study, along with the finding that only 39.6% of respondents were able to correctly differentiate between HIV infection and AIDS, indicating a substantial gap in fundamental HIV-related knowledge. Although 76.6% of participants correctly identified the primary modes of HIV transmission, persistent misconceptions regarding casual transmission highlight the need for more comprehensive educational interventions. These findings emphasize the importance of targeted educational campaigns aimed at correcting misconceptions and addressing prevalent myths about HIV transmission (19, 28, 29).

Notably, 44.0% of participants continued to perceive HIV as a fatal disease, despite 62.5% acknowledging that it can be effectively managed with treatment. This discrepancy suggests the presence of significant emotional barriers that may transform factual medical knowledge into fatalistic beliefs, thereby perpetuating fear and discouraging proactive health-seeking behaviors. Furthermore, the finding that only 47.8% of participants recognized condom use as a preventive method is particularly concerning, as it highlights a critical gap in comprehensive sexual health education- an area that is often constrained by conservative sociocultural norms. Similar findings, interpretations, and conclusions have previously been reported in studies conducted in other regions of the country as well as abroad (18, 19, 24, 29, 30). Self-assessment of knowledge further illuminates this gap, with respondents reporting nearly equal proportions of “good” (37.3%), “moderate” (35.8%), and “limited” (26.9%) knowledge. This distribution suggests that a substantial portion of the population may overestimate their level of understanding, reinforcing the necessity for structured, objective educational interventions rather than reliance on perceived knowledge alone.

Attitudinal barriers- particularly the pervasive stigma- emerged as a central theme. Notably, 54.3% of participants perceived Saudi society as “not accepting” of people living with HIV (PLWHIV), whereas only 3.2% considered it “fully accepting.” Such deeply rooted stigma has been documented in the literature, where HIV is often associated with “improper practices” or perceived as divine punishment (7, 21, 25). These misconceptions and beliefs contribute to an environment characterized by fear and isolation, which can directly affect testing behaviors and access to care. Furthermore, the high proportion of participants who consider discussing HIV to be a culturally and religiously sensitive topic further exacerbates this challenge, fostering societal silence that limits open dialog and constraints comprehensive public health education. The observation that only 20.6% of respondents believed families provide adequate support to PLWHIV, while nearly half (46.8%) reported being “not sure,” reflects a lack of confidence in essential social support systems, Family and social support are critical determinants of psychological well-being, treatment adherence, and disclosure among PLWHIV. Previous studies have similarly demonstrated that perceived stigma and limited family support can discourage disclosure and negatively affect the overall well-being of PLWHIV (31, 32).

Low rates of voluntary HIV testing uptake remains a critical and significant public health concern globally, as early diagnosis is essential for timely initiation of treatment and prevention of onward transmission. In the present study, a considerable proportion of participants (68.4%) reported that they had never undergone HIV testing. This finding is broadly consistent with recent research conducted in Saudi Arabia by Alshahrani and colleagues in 2025, which highlighted a generally low perception of personal risk for HIV infection among Saudi population, potentially contributing to reduced testing behavior (33). The present study further explored reasons for not undergoing HIV testing. The most commonly reported reason was the belief that testing was unnecessary (76.3%), indicating a low perceived susceptibility to HIV infection. This perception reflects an important gap in awareness regarding universal vulnerability to HIV and the benefits of early diagnosis. Similar findings have been reported in other studies, were low perceived risk and limited knowledge about HIV transmission and prevention were identified as major determinants of low testing uptake (33, 34). Additional barriers identified in this study included lack of knowledge about where HIV testing services are available (17.2%) and lack of time (15.7%), suggesting that both informational and logistical factors may hinder access to testing services. Although reported less frequently, fear of negative reactions from family members of the community (4.0%) highlights the continuing role of HIV-related stigma. Previous literature have consistently identified stigma and fear of social judgment as significant obstacles to HIV testing and disclosure, particularly in conservative sociocultural contexts (35, 36). Encouragingly, more than half of the participants who had never been tested (54.5%) indicated that they would be willing to undergo HIV testing if it were offered free of charge and ensured confidentiality. This finding suggests that expanding accessible, confidential, and community-friendly testing services could substantially improve testing uptake. Integrating routine HIV screening into primary healthcare settings and strengthening public awareness campaigns may therefore represent effective strategies to promote early detection and reduce undiagnosed infections.

The findings of the present study demonstrate encouraging attitudes toward HIV-related public health interventions. A large majority of respondents (93.5%) supported the inclusion of HIV-related information within educational curricula, while 67.6% “strongly agreed” with the acceptance of HIV awareness programs, yielding a high mean agreement score (4.55 ± 0.74). These results suggest a substantial level of societal readiness for structured education initiatives addressing HIV prevention and awareness. Integrating HIV education into formal curricula has been widely recommended as a critical strategy to improve knowledge, correct misconceptions, and reduce stigma surrounding the disease. Previous research as shown that improved knowledge about HIV transmission and prevention is significantly associated with more positive attitudes toward PLWHIV and reduced stigmatizing perceptions (37). On another note, the relatively high proportion of participants who aware of simple and accurate laboratory tests for HIV (66.9%) indicates a moderate level of awareness regarding diagnostic services. Awareness of testing options is a crucial determinant of early diagnosis and timely treatment initiation. Studies demonstrated that individuals with higher knowledge levels about HIV testing and prevention are more likely to exhibit favorable attitudes toward testing and preventive measures. Conversely, limited knowledge and misconceptions have been linked to stigmatizing attitudes and reluctance to engage with HIV-related health services (38).

The finding that 63.4% of respondents supported random HIV testing in public places further reflects openness toward expanding testing strategies and improved accessibility. Public health literature consistently emphasizes that increasing the availability of testing services is a key component of HIV control programs. Expanded testing initiatives, including community-based and venue-based testing, have been shown to reach populations that may not otherwise access traditional healthcare facilities and can significantly improve early detection rates. In addition perceptions regarding anonymous rapid HIV resting highlight the importance of confidentiality in overcoming barriers to testing. In the present study, a combined proportion of participants (38.1% strongly agree and 26.5% agree) believed that anonymous rapid HIV testing could help reduce stigma. Concerns about confidentiality and fear of social judgment have been identified as major factors discouraging individuals from seeking HIV testing. Evidence suggests that stigma and concerns about privacy significantly influence individual’s willingness to undergo HIV testing even when services are available (39). Similarly, the finding that 70.9% of respondents believed broader testing availability would increase testing uptake further reinforces the perceived importance of accessibility and supportive testing environments. Prior research has indicated that stigma, fear of discrimination, and social repercussions remain important barriers to HIV testing in may settings, despite the availability of testing service (40). Therefore, interventions that normalize testing, ensure confidentiality, and integrate HIV testing into routine healthcare or community settings may contribute to increased testing uptake and earlier diagnosis. Overall, these perceptions reflect a growing public recognition of practical and feasible strategies to address existing barriers related to HIV testing and stigma. The strong support for educational interventions, expanded testing availability, and anonymous testing options highlights the potential for implementing comprehensive public health strategies aimed at improving HIV awareness, reducing stigma, and enhancing early detection and prevention efforts.

The inferential analyses conducted in the present study provide important insights into the demographic and behavioral factors associated with HIV knowledge and testing practices. A statistically significant association was observed between educational level and HIV knowledge (*p* = 0.003), with individuals holding postgraduate qualifications demonstrating the highest knowledge scores. This is consistent with global evidence indicating that higher educational attainment is strongly associated with improved health literacy and greater understanding of HIV transmission, prevention, and treatment. Formal education plays a critical role in disseminating accurate health information and fostering critical thinking skills that enable individuals to access and interpret health-related knowledge effectively. Previous studies have similarly reported that individuals with higher education levels tend to demonstrate significantly better knowledge about HIV and are more likely to adopt preventive health behaviors compared with those with lower educational attainment (41, 42). Interestingly, gender did not demonstrate a statistically significant difference in HIV knowledge in this study. This suggests that access to information and awareness about HIV may be relatively similar across male and female participants within the studied population. Comparable findings have been reported in several population-based studies where gender differences in HIV knowledge were minimal once access to education and health information sources was comparable. However, other studies conducted in different sociocultural contexts have reported gender disparities in HIV knowledge, often influenced by differences in educational opportunities and access to health information (43, 44).

A particularly notable finding of this was the strong association between previous HIV testing and knowledge levels (*p* < 0.001). Participants who had previously undergone HIV testing demonstrated significantly higher knowledge scores (7.94 ± 2.04) compared to those who had never been tested (6.01 ± 2.37). This finding suggests a potentially bidirectional relationship between knowledge and testing behavior. On one hand, individuals with higher levels of HIV-related knowledge may be more motivated to seek testing due to increased awareness of risk factors and the benefits of early diagnosis. On the other hand, the process of undergoing testing often involves pre-test and post-test counseling, which may contribute to increased knowledge and improved understanding of HIV prevention and transmission. Previous research has similarly demonstrated that HIV testing services, particularly when accompanied by counseling, play a significant role in improving individuals’ knowledge and risk perception (45). Furthermore, the present study identified a strong association between receiving health education and higher HIV knowledge scores (*p* < 0.001). Participants who reported receiving health education exhibited substantially higher testing rates compared with those who had not received such education (62.0% vs. 29.5%). This finding strongly supports the effectiveness of targeted health education initiatives in improving both knowledge and health-seeking behaviors related to HIV. Health education programs are widely recognized as essential components of HIV prevention strategies, as they help correct misconceptions, reduce stigma, and promote proactive health behaviors such as voluntary counseling and testing. Evidence from multiple international studies indicates that structured HIV education programs significantly increase individuals’ willingness to undergo testing and adopt preventive behaviors (46).

Taken together, these findings underscore the critical role of education-both formal and health-specific- in shaping HIV knowledge and testing behaviors. Educational attainment appears to facilitate greater understanding of HIV-related issues, while targeted health education programs further enhance knowledge and encourage engagement with preventive health services such as HIV testing. These results highlight the importance of strengthening educational interventions and expanding public health education initiatives to improve HIV awareness and increase testing uptake within the population. The associations between HIV testing history and education level, perception of societal acceptance, and health education further reinforce these points. Higher education and health education exposure were linked to higher testing rates. Interestingly, more previously tested individuals perceived society as “non-accepting” (66.0% vs. 48.9% among never tested). This counterintuitive finding might suggest that individuals who have undergone testing have a more realistic understanding of societal stigma, possibly having experienced or witnessed it firsthand, or that the fear of stigma drives their decision to seek testing confidentially. This highlights the double-edged sword of stigma: it deters some from testing, but for others, it might reveal the necessity of discreet testing.

Conclusively, the study findings further strengthen the importance of culturally appropriate public health interventions. Sociocultural beliefs, stigma, and misconceptions related to HIV may continue to influence HIV knowledge and testing behaviors despite higher educational attainment among participants. Therefore, culturally sensitive awareness campaigns, community-based education programs, and stigma reduction strategies that align with local cultural and religious values are essential to improve HIV knowledge, promote positive attitudes toward testing, and increase the uptake of HIV testing services.”

## Limitations

This study has several limitations that considered when interpreting the findings. First, the use of convenience-based sampling may have introduced selection bias, as participants were recruited based on accessibility and willingness to participate. Although this approach enabled data collection from different regions of Saudi Arabia, the sample may not fully represent the broader Saudi adult population, thereby limiting the generalizability of the findings. In particular, individuals with higher educational attainment or greater interest in health-related topics may have been overrepresented. Second, the study relied on self-reported data regarding HIV knowledge and testing behavior, which may be subject to recall bias and social desirability bias. Participants may have over-reported socially acceptable behaviors or underreported sensitive attitudes and practices related to HIV testing due to cultural and social sensitivities surrounding the topic. Third, the cross-sectional design of the study limits the ability to establish causal relationships between variables. While associations between HIV knowledge and testing behavior were identified, the temporal direction of these relationships cannot be determined. Despite these limitations, the study provides valuable insights into HIV knowledge and testing behaviors among adults in Saudi Arabia and highlights the need for culturally appropriate public health interventions and further research using more representative sampling methods and longitudinal designs.”

## Conclusion

The findings of this study necessitates the pressing need for strengthened, comprehensive and culturally appropriate public health strategies to improve HIV awareness, mitigate pervasive stigma, and promote voluntary HIV testing in Saudi Arabia. The findings also emphasize that while formal education and health education campaigns are effective in improving knowledge and testing rates, they must be complemented by strategies that directly confront and dismantle the deeply ingrained attitudinal barriers. Public support for increased HIV education in curricula, widespread awareness programs, and accessible confidential testing modalities presents a unique opportunity for policymakers and health authorities. By leveraging these positive public sentiments and addressing the identified knowledge deficits and societal stigmas, Saudi Arabia can make significant strides toward achieving the UNAIDS 95–95-95 targets, fostering a more informed, empathetic, and health-conscious society.

## Data Availability

The original contributions presented in the study are included in the article/supplementary material, further inquiries can be directed to the corresponding author.
